# The impact of AI service quality on researchers’ work wellbeing: a dual-path moderated mediation model

**DOI:** 10.3389/fpsyg.2026.1946487

**Published:** 2026-10-16

**Authors:** Shuyang Liu, Tongyao Liu

**Affiliations:** 1 Office of Humanities and Social Sciences, Yangzhou University, Yangzhou, China; 2 School of Media, Film and Television, Jiangsu Normal University, Xuzhou, China

**Keywords:** AI service quality, AI trust, cognitive verification load, JD-R model, research self-efficacy, work wellbeing

## Abstract

As AI tools permeate scientific research, a critical question emerges: does better AI actually make researchers happier? Grounded in the Job Demands-Resources (JD-R) model, this study proposes and tests a dual-path moderated mediation framework in which perceived AI service quality influences researcher work well-being through two parallel psychological routes. The motivational gain pathway posits that higher quality is positively related to research self-efficacy, while the loss-alleviation pathway posits that higher quality is negatively related to cognitive verification load. AI trust is introduced as a moderator of both first-stage paths. Using data from 439 researchers and PROCESS macro models 4 and 7, we found that AI service quality did not directly affect well-being (*β* = 0.07, *p* = 0.260), but exerted a fully mediated total effect. The loss pathway via cognitive verification load reduction was the sole significant mediating mechanism (indirect effect = 0.08, 95% CI [0.028, 0.135]). AI trust significantly moderated only the loss pathway (*β* = −0.21, *p* < 0.001): the cognitive-relief effect became significant only at medium and high trust levels, remaining inoperative for low-trust researchers. These findings reframe the value of AI quality in research settings as cognitive offloading rather than competence enhancement, and position trust as a threshold condition rather than a continuous amplifier of AI benefits.

## 1 Introduction

The integration of AI tools into scientific research has accelerated dramatically. In this study, AI refers to generative artificial intelligence, which is an artificial intelligence system capable of generating new content (such as text, code, and analysis) based on learned patterns (Feuerriegel et al., 2024), as it is this class of tools that has most rapidly penetrated scientific workflows. Large-scale surveys confirm AI has evolved from analytical assistants to collaborative partners for hypothesis generation, experimental design and manuscript drafting, greatly boosting research efficiency in data processing and literature mining (Van Noorden and Perkel, 2023). Yet a critical paradox persists: despite AI’s functional advantages, 82% of scientists report reduced job satisfaction post-AI adoption (Toner-Rodgers, 2024). This counter-narrative likely reflects a distinctive feature of scientific work: researchers derive identity and meaning not just from outputs, but from the intellectual process itself. When AI intrudes into that process—even competently—it may erode the sense of personal achievement and cognitive ownership that underpins well-being. This tension between AI as resource and AI as threat to self-concept is the motivating paradox of the present study.

We address this paradox through the lens of the Job Demands-Resources (JD-R) model (Bakker and Demerouti, 2007), which holds that work-environment features operate through two distinct psychological processes: a motivational process driven by resources, and a health-impairment process driven by demands. In this model, AI service quality is treated as a technological job resource, whereas cognitive verification load is treated as a cognitive job demand. As a resource, high-quality AI may strengthen researchers’ confidence in their scientific capabilities—their research self-efficacy. As a demand-reducer, it may lower the cognitive burden of validating AI outputs—their cognitive verification load. These two pathways are not mutually exclusive; they may operate simultaneously but with different strengths, conditionalities, and practical implications. A critical boundary condition for both pathways is trust in AI. Cognitive offloading requires willingness to rely on AI outputs; competence enhancement requires willingness to internalize AI-assisted successes as self-attributed. Both forms of benefit may therefore be gated by trust.

The existing literature points out several key limitations that this study needs to address. First, prior JD-R applications to technology work treat digital tools as uniform resources, failing to unpack dual parallel psychological pathways through which AI service quality shapes well-being: a motivational gain path via enhanced self-efficacy, and a health-loss alleviation path via reduced cognitive verification load. Most prior work only tests a single mediating mechanism, ignoring the coexistence and relative dominance of competence enhancement vs. cognitive offloading in academic research contexts. Second, studies of AI trust treat trust as a universal linear amplifier for all technology benefits, without examining its asymmetric moderating effect across distinct psychological mechanisms. It remains untested whether trust equivalently strengthens both gain and loss pathways, or only gates one route as a threshold condition. Third, mainstream AI service quality research prioritize productivity and behavioral adoption outcomes, rarely centering researcher-specific work well-being and the unique cognitive verification load unique to academic AI use.

This study makes three principal contributions. First, it provides a theoretically grounded dual-pathway account of how AI quality affects researcher well-being, extending the JD-R model to AI-augmented scientific work. Second, it empirically distinguishes the relative strength of the gain versus loss pathways, revealing which mechanism dominates and under what conditions. Third, it identifies an asymmetric moderating role of AI trust—active in the loss pathway but absent in the gain pathway—offering a nuanced account of the psychological mechanisms through which trust generates well-being benefits in human-AI collaboration.

### 1.1 Alignment with motivational functioning and educators’ professional wellbeing

As university researchers and academic educators, scholars represent a core group within educational settings whose long-term occupational engagement hinges on stable motivational resources and balanced psychological states. Previous academic research has extensively explored how workplace resources and contextual demands shape educators’ motivational pathways and overall occupational wellbeing (Schaufeli and Taris, 2013a; Schaufeli and Taris, 2013b; Skaalvik and Skaalvik, 2018), yet few studies have extended the JD-R model to academic environments integrated with artificial intelligence (Dixit and Upadhyay, 2021; Xu and Hu, 2024), as new technological factors reshape educators’ cognitive load and efficacy beliefs. Our dual-path model fills this gap: the motivational gain pathway reflects educators’ competence-based motivational functioning via research self-efficacy (a key academic belief), while the loss-alleviation pathway captures demand-driven psychological strain that disrupts educational professionals’ wellbeing. By centering academic educators’ daily AI-assisted work experiences, this paper extends the literature on educational motivational functioning to contemporary digital research contexts and delivers targeted insights into educators’ professional well-being under AI penetration.

## 2 Theoretical background and hypotheses

### 2.1 Perceived AI service quality and researcher work wellbeing

Service quality is fundamentally a subjective comparative judgment—users’ perception of whether a service meets or exceeds their expectations (Parasuraman et al., 1988). In AI contexts, this perception encompasses multiple dimensions: technical reliability, responsiveness, ease of use, personalization, and safety (Noor et al., 2022). Wixom and Todd (2005) advanced the field by integrating user satisfaction with technology acceptance models, emphasizing that evaluations of the system itself and of one’s use behavior are conceptually distinct but jointly important.

For the present study, perceived AI service quality in research contexts is defined as researchers’ overall subjective assessment of the effectiveness, reliability, and value of AI tools in supporting their research activities. Researcher work well-being is operationalized as a multidimensional construct encompassing the positive affect, meaning, achievement, and psychological functioning experienced in scientific work (Ryff, 1989; Zheng et al., 2015). Critically, well-being in this context extends beyond satisfaction to include researchers’ sense of purpose, growth, and authentic engagement with scientific inquiry.

We do not hypothesize a direct effect of AI service quality on well-being. Instead, consistent with the JD-R model’s emphasis on process-mediated pathways, we propose that quality operates exclusively through two psychological mechanisms, which are elaborated below.

### 2.2 The job demands-resources framework

The Job Demands-Resources (JD-R) Model was initially proposed by Demerouti and colleagues in 2001, aiming to provide a more universal theoretical perspective for explaining job burnout (Demerouti et al., 2001). The core assumption of this model is that the characteristics of any work environment can be categorized into two main types: job demands and job resources. Job demands refer to aspects of work that require continuous physical, cognitive, or emotional effort, such as workload, time pressure, or emotional requirements. These demands can drain employees’ energy and may lead to fatigue, exhaustion, and other health-related issues (Xanthopoulou et al., 2007). In contrast, job resources refer to physical, psychological, social, or organizational factors that help achieve work goals, reduce job demands, or promote personal growth and development, such as autonomy, social support, feedback, or skill variety. These resources can stimulate employee motivation and enhance work engagement and performance.

In their 2004 study, Schaufeli and Bakker found through a multi-sample study that job demands primarily predict burnout (especially exhaustion), while job resources primarily predict work engagement (Schaufeli and Bakker, 2004). This establishes the two core psychological processes of the model. The first is the ‘health impairment process,’ where high job demands continuously deplete employees’ energy reserves, potentially leading to chronic stress responses, burnout, and health problems. The second is the ‘motivational process,’ where sufficient job resources can satisfy employees’ basic psychological needs (such as autonomy, competence, and relatedness), thereby enhancing their work engagement, organizational commitment, and performance (Schaufeli and Taris, 2013a; Schaufeli and Taris, 2013b). These two processes are not isolated; they are often interconnected, forming ‘gain paths’ or ‘loss paths’ (Bakker and Demerouti, 2017).

Within the framework of this model, we adopt a consistent construct mapping: perceived AI service quality is conceptualized as a technological job resource, whereas cognitive verification load is conceptualized as a cognitive job demand. As a job resource, high-quality AI tools support goal achievement, help researchers accomplish their research goals, and boost their sense of self-efficacy, thereby activating the motivational gain process. The cognitive verification load arises from the integration of AI into research, which imposes a distinct additional cognitive demand—researchers must verify and correct AI outputs. High-quality AI services are expected to reduce this verification demand by producing more reliable and transparent outputs, whereas lower-quality services leave the demand unrelieved. In this way, perceived AI service quality operates as a job resource that simultaneously fuels the motivational gain pathway and alleviates the health-impairment pathway, while cognitive verification load functions purely as a cognitive job demand within the latter pathway.

We formalize research self-efficacy (gain resource mediator) and cognitive verification load (loss demand mediator) as two parallel psychological pathways linking AI service quality to work well-being. These two parallel pathways are conceptually distinct in their underlying psychological processes. They can operate simultaneously to promote work well-being but may differ in empirical strength and boundary conditions. We focus on these two mediators for several theoretical reasons. First, research self-efficacy is the specific domain manifestation of self-efficacy beliefs in scientific work (Bandura, 1977; Van der Westhuizen, 2014) and functions as a key personal resource that amplifies the motivational process of the JD-R model (Xanthopoulou et al., 2007; Guglielmi et al., 2012). It captures the potential competence gains that reliable AI-assisted research may confer, which generic attitude constructs such as perceived usefulness or job involvement cannot. Moreover, recent scale-development work on AI self-efficacy confirms that users form distinct, domain-specific confidence beliefs about AI-supported performance (Wang and Chuang, 2024). Second, cognitive verification load reflects the unique additional burden of repeatedly verifying AI-generated research outputs—a burden that general technostress or cognitive-overload constructs fail to isolate because it is specific to human-AI research collaboration (Khan and Suhluli, 2025). Third, juxtaposing these two mediators directly addresses the core paradox motivating this study—why high-quality AI does not uniformly improve researcher well-being—by testing, within a single model, whether AI functions as a capability enhancer, a burden reliever, or both, and under what conditions each mechanism operates.

### 2.3 Motivational gain pathway: research self-efficacy as mediator (H1)

Research self-efficacy, as a specific manifestation of self-efficacy theory in the field of scientific research, is rooted in Bandura’s social cognitive theory. This theory posits that self-efficacy is an individual’s belief in their ability to organize and execute the actions required to achieve specific goals, profoundly influencing their behavioral choices, effort levels, and persistence (Bandura, 1977). When this concept is specifically applied to the field of scientific research, research self-efficacy refers to a researcher’s judgment of their own ability to successfully complete various research tasks, such as proposing research questions, designing experiments, analyzing data, and writing papers (Van der Westhuizen, 2014).

Within the JD-R framework, it constitutes a personal resource: a positive self-belief associated with resilience that amplifies the motivational effects of job resources (Guglielmi et al., 2012). When researchers perceive AI tools as reliable and effective, this assessment signals that the tools are capable partners for research work, which may strengthen their confidence in tackling research challenges with AI assistance (Lee et al., 2023). Elevated self-efficacy, in turn, reduces helplessness during research, increases perceived control, and generates greater satisfaction and intrinsic motivation (Elballah et al., 2024). Therefore, within the JD-R framework, this constitutes a personal resource that mediates the model’s motivational gain process: favorable external job resources (high AI service quality) translate into enhanced internal self-beliefs, which drive greater intrinsic motivation and work wellbeing.

*H1*: AI service quality has a positive indirect effect on work well-being through research self-efficacy. Higher AI service quality is positively associated with research self-efficacy, which in turn is positively associated with work wellbeing (Motivational Gain Pathway).

### 2.4 Loss-alleviation pathway: cognitive verification load as mediator (H2)

Cognitive verification load refers to the mental effort required to check, cross-validate, and correct AI outputs—a demand that has become increasingly salient as concerns about AI hallucinations and systematic errors have grown. Drawing on cognitive load theory (Sweller, 1988), this load functions as an extraneous demand in the JD-R sense: it consumes cognitive resources without directly advancing research goals. Cognitive verification load is defined operationally as the unique subtype of extraneous cognitive burden incurred exclusively by academic researchers to repeatedly examine, cross-check, fact-check, correct logical flaws, and identify hallucinations or computational errors in AI-generated research texts, datasets, figures, and inference arguments during research work. Operationally, it captures the continuous mental effort spent on verifying AI outputs that researchers did not personally generate, separate from the cognitive resources invested in original hypothesis deduction, experimental design or independent data analysis. It differs fundamentally from the broad cognitive load construct from cognitive load theory. General cognitive load covers intrinsic task complexity load and general presentation-induced extraneous load across all intellectual work (Sweller, 2011), whereas cognitive verification load is a context-bound subcategory of extraneous load that only emerges in human-AI research collaboration. Unlike cognitive overload, a state in which total working-memory demand exceeds available capacity, cognitive verification load is a specific, recurring source of demand tied to the epistemic uncertainty of AI-generated outputs. It is also conceptually related to, yet distinct from, cognitive offloading. Offloading is the strategy of delegating cognitive work to external tools to reduce internal demand (Risko and Gilbert, 2016), whereas cognitive verification load is the residual burden of monitoring and correcting the outputs of such offloaded work. The core lies in repeatedly checking the uncertain outputs of AI, rather than the mental effort of coming up with original research ideas. Therefore, we believe that cognitive verification load is extremely important for researchers in this intelligent era.

Research indicates that high-quality AI output can help users build accurate mental models more quickly, enabling them to more effectively determine when to trust the AI and when to intervene and verify, rather than blindly repeating checks (Bansal et al., 2019). High-quality service reduces the uncertainty and ambiguity of information, making the verification process easier. Conversely, if the perceived quality of AI services is low, researchers need to invest more cognitive resources to question, check, and correct the AI’s outputs, thereby bearing a higher verification burden (Bremnes et al., 2024). In other words, when the quality of AI services is higher, and its outputs are more reliable and explanations clearer, the cognitive verification burden that researchers need to invest in validating AI results will be relatively lower. The increase or decrease in cognitive load directly affects workers’ well-being. On one hand, when researchers handle multiple new tasks simultaneously or work in high-pressure environments, cognitive demands increase sharply. This can easily lead to mental fatigue and increased stress, thereby reducing overall well-being and job satisfaction (Meyer and Hünefeld, 2018). On the other hand, a moderate cognitive load allows researchers to invest more cognitive energy into creative scientific thinking, improving efficiency and autonomy, thereby enhancing well-being. Cognitive verification load is conceptualized as an extraneous cognitive job demand under the JD-R model, rather than a form of resource.

*H2*: AI service quality has a positive indirect effect on work well-being through reduced cognitive verification load. Higher AI service quality is negatively associated with cognitive verification load, which in turn is negatively associated with work well-being (Loss-Alleviation Pathway).

### 2.5 The moderating role of AI trust

Trust in AI is the willingness to depend on an AI system under conditions of uncertainty, reflecting positive expectations about its behavior (Afroogh et al., 2024). It constitutes a psychological lens through which service quality signals are interpreted and acted upon.

It is important to conceptually distinguish AI trust from AI service quality. Perceived AI service quality represents users’ evaluative judgment of the AI system’s actual performance attributes (including reliability, effectiveness and safety), centering on what the AI system delivers (Noor et al., 2022). By contrast, AI trust is a psychological state of the researcher: willingness to rely on AI outputs under uncertainty and subjective expectations about AI performance (Fischer and Schellenbach, 2025). Quality is a performance-oriented appraisal of system behavior; trust reflects a user-side readiness to depend on the system. Researchers may perceive high AI service quality based on observed performance yet maintain low trust and refuse unguarded reliance on AI-generated research content. While service quality may be related to trust, the two constructs remain theoretically distinct. The data reported in Section 3 supplement this theoretical distinction, with the Fornell-Larcker criterion and HTMT ratio supporting discriminant validity.

When researchers perceive AI services as high-quality, this perception itself may directly boost their confidence in using AI tools to complete research tasks. The extent and effect of this positive impact largely depend on the researchers’ level of trust in AI. Studies have shown that trust in AI can moderate the relationship between an individual’s confidence in the technology and their behavioral intentions (Tao et al., 2025). In the gain pathway, high-trust researchers may be more receptive to internalizing positive AI performance as evidence of their own enhanced research capability, amplifying the self-efficacy signal from quality (Obenza et al., 2024). Conversely, distrustful researchers may attribute AI successes to the tool rather than to themselves, attenuating the competence-enhancing effect.

*H3*: AI trust positively moderates the gain pathway, such that the correlation of AI service quality on research self-efficacy is stronger at higher trust levels.

In the loss pathway, trust directly regulates the willingness to rely on AI outputs without extensive verification. High-trust researchers are more willing to accept outputs as sufficiently reliable, enabling cognitive offloading and thereby amplifying the load-reduction benefits of high quality (Wang, 2021). Low-trust researchers maintain sustained vigilance irrespective of quality, nullifying the cognitive-relief potential of even high-quality service (Dietvorst et al., 2015) (Figure 1).

**Figure 1:**
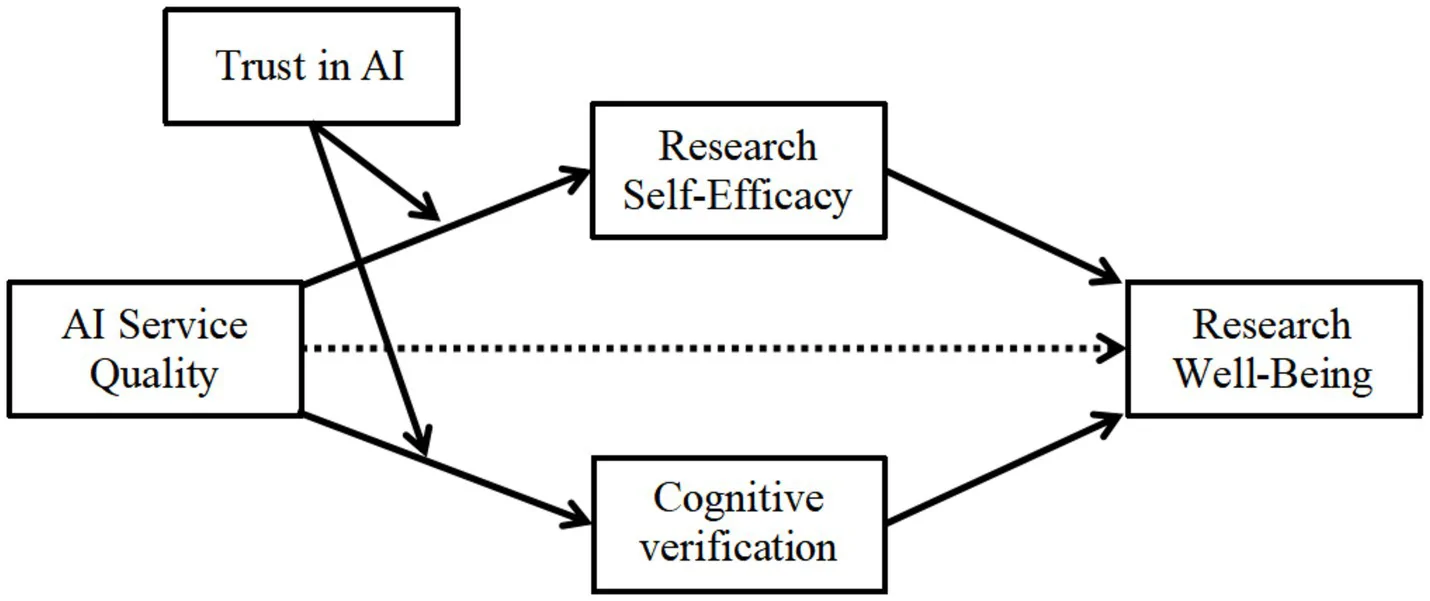
Dual-path moderated mediation model. Solid arrows = hypothesized effects. Dashed arrow = direct path (expected non-significant).

*H4*: AI trust negatively moderates the loss pathway, such that the correlation of AI service quality on cognitive verification load is stronger (more negative) at higher trust levels.

## 3 Method

### 3.1 Measures

We used five established measurement scales to conduct cross-cultural adaptation for Chinese researchers according to the forward backward translation method. First, two bilingual psychological researchers independently translated the English scale into Chinese. Next, two other professional translators translated the preliminary Chinese version back to English, but could not use the original scale. Then, the research team compared the back translation version with the original scale, discussed the inconsistent items, and modified the wording to ensure language consistency.

Subsequently, we invited five researchers and two psychologists to assess the cultural suitability of each project. In order to conform to the daily context of Chinese researchers, several expressions are fine tuned on the premise of retaining the original structural meaning. Finally, the validity of the questionnaire was verified by factor analysis and model fitting evaluation. During the formal investigation, we also tested the reliability and validity of the final data.

#### 3.1.1 AI service quality

AI service quality was assessed using an adapted version of the AISAQUAL scale (Noor et al., 2022). The original scale was developed for customer service AI contexts. We revised it to fit scientific research scenarios by retaining three conceptually aligned core dimensions—system reliability, functional responsiveness, and research value—and removing dimensions specific to customer service settings. After revision, the scale has three dimensions, each with three items, for a total of 9 items, and uses a 7-point Likert scale for scoring. Cronbach’s *α* = 0.887. It performed well in the confirmatory factor analysis (X^2^/df = 2.82, CFI = 0.98, TLI = 0.97, RMSEA = 0.06, SRMR = 0.04). The AVE values for the three dimensions were 0.53, 0.67, and 0.77, with CR values of 0.77, 0.86, and 0.91, respectively, indicating good convergent validity. The factor load value of all items exceeds 0.5. The square root of AVE of each factor is greater than the maximum value of the correlation coefficient between this factor and other factors, indicating that it has good discriminant validity.

#### 3.1.2 AI trust

Trust was measured using the Epistemic Trust in Generative AI for Higher Education Scale (ETGAI-HE; Pandey et al., 2025). After being revised, this scale includes two sub-dimensions and 9 items. The second item needs to be reverse scored. Cronbach’s *α* = 0.892. The confirmatory factor analysis showed good fit indicators (X^2^/df = 4.10, CFI = 0.96, TLI = 0.95, RMSEA = 0.08, SRMR = 0.05). The AVE values for the two dimensions were 0.58, 0.51, with CR values of 0.86, 0.81. The factor load value of all items exceeds 0.5. The square root of the AVE of each factor is greater than the maximum correlation coefficient between that factor and any other factor, indicating that it has good discriminant validity.

Given that the concepts of AI Service Quality and AI Trust may be confused, we also use the Fornell Larcker criterion and HTMT ratio for testing. The results showed that the AVE square root of AI Service Quality (0.834) and AI Trust (0.720) were both greater than the correlation coefficient between the two constructs (0.526). The HTMT value is 0.589, which is below the strict threshold of 0.85. This indicates that the two constructs have good discriminant validity.

#### 3.1.3 Research self-efficacy

The construct of research self-efficacy was drawn from Gurganus (2019), a work-in-progress conference paper adopting research-self-efficacy measures for STEM scholars. We adapted well-established research self-efficacy item stems to the context of AI-assisted scientific research. This scale has a total of 11 items and the first four items require reverse scoring. Cronbach’s *α* = 0.852. Its confirmatory factor analysis showed good fit indicators (X^2^/df = 3.20, CFI = 0.95, TLI = 0.94, RMSEA = 0.07, SRMR = 0.05). The AVE values for the two dimensions were 0.51, 0.50, with CR values of 0.80, 0.87. The factor load value of all items exceeds 0.5. The square root of the AVE of each factor is greater than the maximum correlation coefficient between that factor and any other factor, indicating that it has good discriminant validity.

#### 3.1.4 Cognitive verification load

The Cognitive Load Scale (Leppink and Van den Heuvel, 2015) measured intrinsic and extraneous cognitive load in the context of verifying AI outputs. The items used in this study are based on the theoretical framework and scale paradigm of this scale, with the questions being original situational adaptations. All items require reverse scoring (4 items). Higher scores indicate greater load. Cronbach’s *α* = 0.862. This scale has good validity and the model fits quite well (X^2^/df = 1.93, CFI = 0.99, TLI = 0.99, RMSEA = 0.05, SRMR = 0.01). The AVE and CR of the scale were 0.64 and 0.87, respectively. The factor load value of all items exceeds 0.5.

#### 3.1.5 Work wellbeing

Work wellbeing was measured with nine context-adapted items from the UWES-9 (Schaufeli and Bakker, 2010). The original UWES-9 assesses three dimensions: vigor, dedication, and absorption. The internal consistency coefficient of this scale is very high (Cronbach’s *α* = 0.911). The confirmatory factor analysis performed well (X^2^/df = 4.13, CFI = 0.97, TLI = 0.96, RMSEA = 0.08, SRMR = 0.04). The AVE values for the three dimensions were 0.85, 0.68, and 0.66, with CR values of 0.92, 0.86, and 0.88, respectively, indicating good convergent validity. The factor load value of all items exceeds 0.5. The square root of AVE of each factor is greater than the maximum value of the correlation coefficient between this factor and other factors, indicating that it has good discriminant validity.

### 3.2 Participants and procedure

We adopted a combination of convenience sampling and snowball sampling: the links of the online questionnaire survey were distributed through e-mail, chat software and the author’s personal academic network, and the participating researchers were also invited to forward the questionnaire to qualified colleagues. Strict inclusion criteria were set: (1) currently engaged in formal scientific research work at universities or research institutes; (2) have used generative or analytical AI tools for research tasks at least once within the past 12 months. Exclusion criteria covered respondents with less than one AI research use experience, incomplete questionnaire answers, and obvious repetitive/abnormal filling patterns. All participants were affiliated with academic institutions across mainland China; no cross-national samples were included. A small digital gift card reward was provided as participation incentive for fully completed questionnaires.

A total of 500 questionnaires were sent out, and 470 data were finally collected (the response rate was 94%). After excluding questionnaires with abnormal data, 439 valid responses were obtained: 228 men (51.9%) and 211 women (48.1%). Most participants held doctoral degrees (73.6%) and reported 5–15 years of research experience (61.0%). Academic ranks comprised lecturers (52.6%), associate professors (32.6%), and full professors (14.8%). Disciplines covered humanities and social sciences (46.0%) and natural sciences (44.2%), with smaller representation from applied technology and management. All participants were informed of the study’s anonymous nature prior to responding. Table 1 presents full demographic details.

**Table 1:** Participant demographic characteristics (*N* = 439).

**Variable**	**Category**	***n***	**%**
Gender	Male	228	51.9
	Female	211	48.1
Age	<30	25	5.7
	30–39	241	54.9
	40–49	126	28.7
	50–59	43	9.8
	>60	4	0.9
Degree	Bachelor’s	6	1.4
	Master’s	110	25.1
	Doctoral	323	73.6
Rank	Lecturer	231	52.6
	Associate Professor	143	32.6
	Full Professor	65	14.8
Discipline	Humanities and Social Sci.	202	46.0
	Natural Sciences	194	44.2
	Applied Technology	21	4.8
	Management and Arts	22	5.0
Experience	<5 years	69	15.7
	5–15 years	268	61.0
	16–26 years	76	17.3
	>26 years	26	5.9

### 3.3 Analytic strategy

Descriptive statistics and bivariate correlations were computed in SPSS 27.0. Parallel mediation (H1, H2) was tested using PROCESS Model 4 (Hayes, 2018) with 5,000 bootstrap replications and 95% bias-corrected confidence intervals. Moderated mediation (H3, H4) was tested using PROCESS Model 7, which positions AI trust as a first-stage moderator. All models controlled for gender, age, educational degree, academic rank, discipline, and years of research experience. Common method bias was assessed via Harman’s single-factor test (Podsakoff et al., 2003) and full collinearity VIF.

We selected PROCESS Model 7 rather than SEM for three key reasons: (1) Model 7 is tailored to first-stage moderated mediation, directly outputting conditional indirect effects across three moderator levels to test our trust threshold hypothesis; (2) we separately completed all construct validity tests via CFA, so regression-based PROCESS prioritizes clear, uncomplicated interpretation of indirect effect differences without redundant latent model computation; (3) demographic controls can be readily embedded in each regression model, and this analytical method is standard for identical dual-path moderated mediation designs in prior AI occupational research. Although SEM enables joint measurement-structural model estimation, PROCESS better serves our core analytical focus on comparing moderated indirect magnitudes.

## 4 Results

### 4.1 Common method bias and descriptive statistics

Harman’s single-factor test yielded a first-factor variance of 26.94%, below the 40% threshold (Podsakoff et al., 2003), indicating common method bias is not a substantial concern. Following Kock (2015), we conducted full collinearity variance inflation factor (VIF) analysis as an additional diagnostic for common method bias. This test assesses whether shared method variance creates global multicollinearity among all latent constructs in the model. Note that full collinearity VIFs are only reported for endogenous latent variables. AI Service Quality and AI Trust are exogenous predictors without any latent constructs predicting them. The full collinearity VIF values for all endogenous latent variables were: Research Self-Efficacy = 2.567, Cognitive Verification Load = 1.468, and Work Well-Being = 1.210. All values were well below the stringent cutoff of 3.3 for severe common method variance. This result confirms that common method bias did not generate problematic global multicollinearity in our structural model.

Table 2 shows the descriptive statistics and bivariate correlations between all research variables and demographic variables. Mean AI service quality was 4.94 (SD = 0.83), indicating generally positive quality perceptions. AI trust was notably lower (*M* = 3.22, SD = 0.98), suggesting that researchers maintain a guarded epistemic stance toward AI despite positive quality evaluations—a finding that itself hints at the relevance of trust as a moderator. The cognitive verification load was also relatively low (*M* = 3.43, SD = 1.01), consistent with a verification burden that high-quality AI could potentially relieve.

**Table 2:** Descriptive statistics and bivariate correlations among study variables and demographic variables.

**Variable**	***M***	**SD**	**1**	**2**	**3**	**4**	**5**	**6**	**7**	**8**	**9**	**10**	**11**
1. AI service quality	4.94	0.83	—										
2. AI trust	3.22	0.98	0.526***	—									
3. Research self-efficacy	4.45	0.77	0.662***	0.362***	—								
4. Cognitive verification load	3.43	1.01	−0.298***	−0.159***	−0.482***	—							
5. Work wellbeing	4.59	1.00	0.201***	0.078	0.262***	−0.291***	—						
6. Gender	1.48	0.50	−0.061	−0.102*	−0.102*	0.107*	−0.080	—					
7. Age	2.45	0.78	−0.156***	−0.108*	−0.174***	0.066	0.103*	−0.080	—				
8. Degree	2.72	0.48	−0.066	−0.139**	0.034	−0.141**	0.057	−0.175***	0.026	—			
9. Rank	1.62	0.73	−0.069	−0.068	−0.051	−0.030	0.114*	−0.202***	0.540***	0.228***	—		
10. Discipline	1.69	0.78	0.111*	0.132**	0.056	0.025	0.008	−0.100*	−0.123*	−0.122*	0.025	—	
11. Experience	2.13	0.74	−0.148**	−0.082	−0.138**	0.060	0.065	−0.125**	0.759***	0.060	0.566***	−0.089	—

M = mean; SD = standard deviation. *p < 0.05. **p < 0.01. ***p < 0.001.

Bivariate correlations revealed that cognitive load was significantly negatively correlated with all other variables (*p*s < 0.001), while AI trust was not significantly correlated with work wellbeing (*r* = 0.078, *p* > 0.05)—a pattern that foreshadows the moderated, rather than direct, role of trust.

In the correlation of demographic variables, gender had negative significant links with degree (*r* = −0.175, *p* < 0.001), rank (*r* = −0.202, *p* < 0.001), discipline (*r* = −0.100, *p* < 0.05) and research experience (*r* = −0.125, *p* < 0.01). Age showed strong positive correlations with academic rank (*r* = 0.540, *p* < 0.001) and years of research experience (*r* = 0.759, *p* < 0.001), while correlating negatively with discipline (*r* = −0.123, *p* < 0.05) at the moderate significance level. Academic degree was positively associated with rank (*r* = 0.228, *p* < 0.001). Research experience displayed large positive correlations with rank (*r* = 0.566, *p* < 0.001), consistent with career progression logic. Other correlations are not significant. There is also a partial correlation between research variables and demographic variables. Given these significant correlations, all six demographic variables were controlled for in subsequent regression analyses.

### 4.2 Parallel mediation: testing H1 and H2

Results from PROCESS Model 4 are presented in Tables 3, 4. Perceived AI service quality was a significant positive predictor of research self-efficacy (*β* = 0.65, *t* = 17.92, 95% CI [0.537, 0.669], *p* < 0.001) and a significant negative predictor of cognitive verification load (*β* = −0.30, *t* = −6.55, 95% CI [−0.475, −0.256], *p* < 0.001), confirming both first-stage paths. Research self-efficacy positively predicted wellbeing (*β* = 0.14, *t* = 2.08, 95% CI [0.010, 0.348], *p* = 0.038), and cognitive load negatively predicted well-being (*β* = −0.21, *t* = −3.98, 95% CI [−0.309, −0.105], *p* < 0.001). Critically, the direct effect of AI quality on well-being was non-significant (*β* = 0.07, *p* = 0.260), indicating full mediation.

**Table 3:** Parallel mediation model: regression coefficients.

Equation / Predictor	Outcome	*R*	*R* ^2^	*F*	*β*	*t*	LLCI	ULCI	*p*
Equation 1 — Predicting self-efficacy									
AI service quality	Self-efficacy	0.67	0.45	51.03	0.65	17.92	0.537	0.669	<0.001
Gender					−0.06	1.53	−0.200	0.025	0.126
Age					−0.10	−1.71	−0.204	0.014	0.089
Degree					0.06	1.64	−0.020	0.219	0.102
Rank					0.02	0.34	−0.080	0.112	0.738
Discipline					−0.03	−0.69	−0.096	0.046	0.491
Experience					0.01	0.17	−0.107	0.127	0.867
Equation 2 — Predicting cognitive load									
AI service quality	Cog. Load	0.35	0.12	8.69	−0.30	−6.55	−0.475	−0.256	< 0.001
Gender					0.07	1.46	−0.048	0.324	0.146
Age					0.03	0.39	−0.145	0.218	0.696
Degree					−0.13	−2.81	−0.482	−0.086	0.005
Rank					−0.05	−0.80	−0.224	0.095	0.425
Discipline					0.06	1.23	−0.044	0.192	0.220
Experience					0.04	0.58	−0.137	0.251	0.563
Equation 3 — Predicting wellbeing									
AI service quality	Wellbeing	0.36	0.13	7.21	0.07	1.13	−0.061	0.227	0.260
Research self-efficacy					0.14	2.08	0.010	0.348	0.038
Cognitive load					−0.21	−3.98	−0.309	−0.105	< 0.001
Gender					−0.02	−0.40	−0.224	0.148	0.689
Age					0.15	2.15	0.017	0.379	0.033
Degree					0.01	0.26	−0.173	0.225	0.796
Rank					0.05	0.90	−0.086	0.232	0.367
Discipline					0.01	0.25	−0.103	0.133	0.804
Experience					−0.04	−0.59	−0.251	0.135	0.556

Demographic covariates included throughout. LLCI/ULCI = 95% confidence interval limits.

**Table 4:** Bootstrap indirect effect decomposition (5,000 replications).

Effect	Estimate	Boot SE	Boot LLCI	Boot ULCI
Total effect	0.27	0.06	0.154	0.380
Direct effect (c′)	0.08	0.07	−0.061	0.227
Total indirect effect	0.19	0.06	0.058	0.303
Via self-efficacy (gain path)	0.11	0.07	−0.031	0.239
Via cognitive load (loss path)	0.08	0.03	0.028	0.135
Contrast (gain - loss)	0.03	0.08	−0.142	0.194

Numerical value of loss path indicate CI excludes zero. Non-significant direct effect confirms full mediation.

Bootstrap analysis confirmed a significant total indirect effect (Effect = 0.19, 95% CI [0.058, 0.303]). Decomposing this, the self-efficacy path (Indirect Effect 1 = 0.11, 95% CI [−0.031, 0.239]) did not achieve strict significance as its interval marginally included zero. The cognitive load path (Indirect Effect 2 = 0.08, 95% CI [0.028, 0.135]) was significant. The contrast between the two indirect effects was non-significant (0.03, 95% CI [−0.142, 0.194]). Thus H2 is fully supported. Per conventional *α* = 0.05 criteria, we cannot reject the null hypothesis of no indirect association, so H1 is unsupported.

### 4.3 Moderated mediation — gain pathway (H3)

PROCESS Model 7 results for the gain pathway are shown in Table 5. The AI service quality (*β* = 0.59, *p* < 0.001) was significant positive predictor of research self-efficacy. However, The AI trust (*β* = 0.01, *p* = 0.665) does not significantly predict research self-efficacy. Besides, the quality × trust interaction was non-significant (*β* = 0.04, *p* = 0.188), meaning there’s no moderating effect. And the 95% confidence interval for the index of moderated mediation included zero (index = 0.01, 95% CI [−0.008, 0.042]). Conditional indirect effects at low, mean, and high trust were 0.17, 0.18, and 0.20 respectively—statistically indistinguishable. H3 is not supported.

**Table 5:** Moderated mediation: gain pathway (self-efficacy mediator).

Predictor	*β*	SE	*t*	*p*	LLCI	ULCI
AI service quality	0.59	0.04	15.18	<0.001	0.516	0.670
AI trust	0.01	0.03	0.43	0.665	−0.052	0.081
Quality × Trust	0.04	0.03	1.32	0.188	−0.019	0.096
*R*^2^ = 0.46, *F* = 39.92						
Conditional indirect effects:						
Low trust (M – 1 SD)	0.17	0.06			0.054	0.288
Mean trust	0.18	0.06			0.059	0.305
High trust (M + 1SD)	0.20	0.07			0.061	0.328
Index of moderated mediation	0.01	0.01			−0.008	0.042

Outcome = research self-efficacy. Demographic covariates included but not shown.

### 4.4 Moderated mediation — loss pathway (H4)

Table 6 presents results for the loss pathway. AI service quality significantly negatively predicted cognitive load (*β* = −0.36, *p* < 0.001). AI trust alone did not significantly predict cognitive load (*β* = −0.01, *p* = 0.913). Critically, the quality × trust interaction was significant and negative (*β* = −0.21, *p* < 0.001), indicating that higher trust amplified the load-reducing effect of high service quality. This result matches the expectation of AI trust as a moderating variable. The index of moderated mediation was significant (index = 0.05, 95% CI [0.017, 0.092]), confirming moderated mediation. H4 gets support.

**Table 6:** Moderated mediation: loss pathway (cognitive load mediator).

**Predictor**	***β***	**SE**	***t***	***p***	**LLCI**	**ULCI**
AI service quality	−0.36	0.06	−5.60	<0.001	−0.481	−0.231
AI trust	−0.01	0.05	−0.11	0.913	−0.114	0.102
Quality × Trust	−0.21	0.05	−4.36	<0.001	−0.301	−0.114
R^2^ = 0.16, *F* = 9.16						
Conditional indirect effects:						
Low trust (M – 1 SD)	0.04	0.02			−0.005	0.090
Mean trust	0.09	0.03			0.041	0.145
High trust (M + 1 SD)	0.14	0.04			0.066	0.223
Index of moderated mediation	0.05	0.02			0.017	0.092

Outcome = cognitive verification load. Significant conditional indirect effects (CI excludes zero) at mean and high trust only.

Conditional indirect effects revealed a clear trust threshold: the indirect effect was non-significant at low trust (Effect = 0.04, 95% CI [−0.005, 0.090]), became significant at mean trust (Effect = 0.09, 95% CI [0.041, 0.145]), and was strongest at high trust (Effect = 0.014, 95% CI [0.066, 0.223]). In other words, high-quality AI services can enhance researchers’ research well-being by alleviating cognitive verification load only when researchers have a moderate or higher level of trust in AI; under low trust conditions, this indirect mechanism cannot take effect. H4 is fully supported. Figure 2 illustrates this interaction: the simple slope for high-trust researchers is substantially steeper (*β* = −0.56) than for low-trust researchers (*β* = −0.15, ns), visually confirming the gating function of trust in the loss pathway.

**Figure 2:**
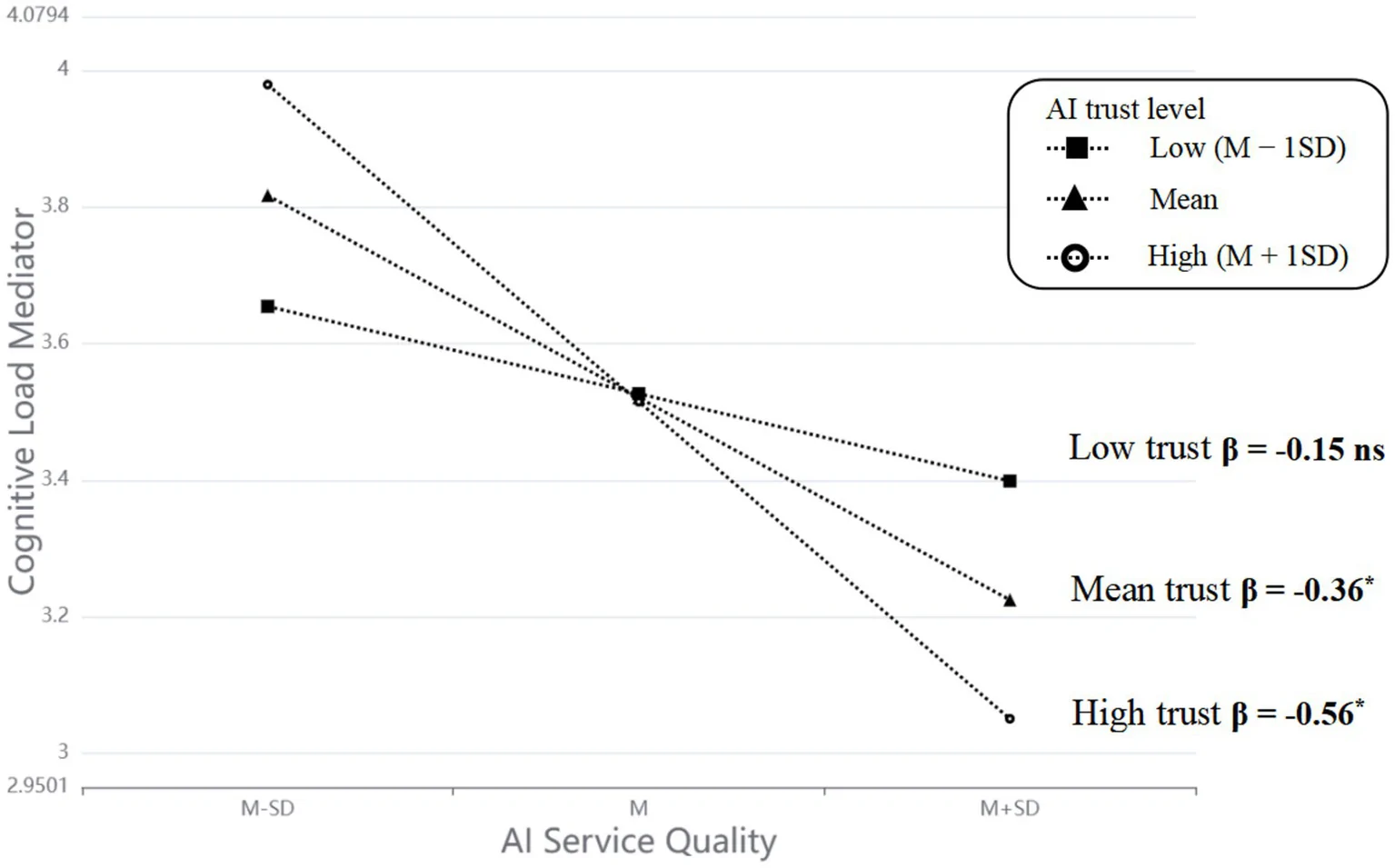
Simple slopes: AI Trust Moderating Quality → Cognitive Load. Simple slopes of AI service quality on cognitive verification load at three levels of AI trust. The ns means that the moderation effect is not significant at low trust levels. *p < 0.05.

## 5 Discussion

This study set out to resolve a fundamental paradox: if better AI tools make research easier, why do so many researchers report lower satisfaction when using AI? Our findings offer a clear structural answer. AI service quality does not improve researcher well-being directly; its benefits are entirely mediated by psychological processes. Moreover, the dominant mediating process is not the one that most technology adoption models would predict—competence enhancement—but rather cognitive burden relief. The following sections unpack the theoretical and practical implications of this pattern.

### 5.1 Cognitive offloading dominates competence enhancement

The loss pathway via cognitive verification load reduction was the only robustly significant mediation pathway. However, the hypothesized self-efficacy gain pathway does not hold. AI service quality is positively correlated with self-efficacy in the first stage of the model, but this correlation has not been transformed into a meaningful indirect correlation with well-being. This asymmetry is theoretically informative. It suggests that in the research context, what AI service quality most reliably delivers to researchers is not empowerment—a stronger sense of their own scientific capability—but relief: a reduction in the exhausting burden of verifying, cross-checking, and correcting AI outputs.

This finding aligns with Routray et al. (2025), who argued that AI’s well-being benefits are most robustly realized through cognitive load reduction, and with Jin et al. (2024), who documented job stress as a mediator between AI threat perception and work wellbeing. It also resonates with the broader observation by Toner-Rodgers (2024) that AI reduces satisfaction in research: if AI primarily relieves verification burden rather than enhancing competence, its effect on the intellectual ownership and identity-affirming aspects of research may indeed be neutral or negative. Better AI does not necessarily make researchers feel more capable; it makes them feel less burdened. These are psychologically distinct outcomes with different implications for well-being, motivation, and professional identity.

Unlike general office employees, researchers derive core self-worth from independent intellectual reasoning, hypothesis construction and critical analysis rather than assisted task completion (Othman and Lo, 2023). Even reliable AI tools raise identity threats: scholars may attribute task success to algorithmic capability instead of their own research talent, weakening the translation of elevated self-efficacy into sustained work well-being. In addition, for academics, the cognitive resources consumed by repeated and mandatory cross-checking of AI outputs far exceed the temporary confidence boost gained from AI-assisted task completion. As a result, the self-efficacy pathway’s positive linkage is too weak to yield a meaningful indirect effect.

This non-significant gain pathway finding also carries important theoretical extensions to the JD-R model. Prior technology-centric JD-R studies often assume resource gains will reliably transmit to well-being outcomes (Lequeurre et al., 2013), yet our data demonstrate this transmission is conditional in research contexts where professional identity is highly tied to intellectual work. The asymmetric strength of the two indirect paths further clarifies that AI’s primary value in academia lies in extraneous demand reduction rather than competence resource accumulation.

### 5.2 Trust as a threshold condition, not a continuous amplifier

The moderating role of trust was asymmetric and threshold-like. Trust moderated the loss pathway but not the gain pathway. In the loss pathway, the cognitive-relief benefit of high service quality was entirely absent at low trust and grew progressively stronger at medium and high trust. This pattern is best characterized not as linear amplification but as a threshold effect: below a critical trust level, the cognitive-relief mechanism simply does not activate, regardless of how high service quality is perceived to be. Researchers who distrust AI maintain persistent verification vigilance as a precautionary posture, nullifying the cognitive savings that quality would otherwise enable.

By contrast, trust did not moderate the quality-to-self-efficacy relationship—suggesting that researchers evaluate their own research capabilities through the direct signal of what AI tools can do for them, rather than through a trust-mediated reframing of that signal. This implies that self-efficacy attributions in AI-augmented research may be more cognitively direct than previously theorized: researchers notice that AI performs reliably and update their task confidence accordingly, irrespective of their general disposition toward the technology.

The asymmetry between pathways extends the emerging literature on trust in AI (Afroogh et al., 2024) by specifying that trust generates well-being benefits primarily through a behavioral-reliance mechanism—reducing vigilance and enabling cognitive offloading—rather than through an identity-enhancing mechanism that inflates competence beliefs. This positioning also enriches the research dimensions of human-machine trust theory, providing empirical evidence for subsequently distinguishing the forms of trust in different psychological pathways. In particular, many researchers simultaneously hold positions in education. AI trust constitutes a unique technological belief that shapes the boundary condition of wellness benefits, complementing existing literature on educators’ belief-based psychological processes in educational workplace contexts. The actionable practical guidance we provide also offers targeted strategies to maintain university researchers’ stable motivational functioning and professional wellbeing.

### 5.3 The bounded resource value of AI quality

Taken together, these findings reframe what it means for AI service quality to be ‘valuable’ in research settings. From a JD-R perspective, the dominant value of quality lies in its demand-reducing function—lowering the extraneous cognitive load of verification—rather than its resource-building function—strengthening self-efficacy. This has a practical implication: there may be a ceiling on the well-being returns from pure quality improvements if trust remains low, because the primary psychological mechanism through which quality benefits researchers cannot activate without a baseline level of trust.

This insight also speaks to a deeper tension in AI-augmented research. The researcher’s intellectual role—formulating questions, evaluating evidence, interpreting findings—is precisely where personal competence is most valued and where identity is most invested (Yar et al., 2025). AI’s most visible intrusions into this space (e.g., generating hypotheses, drafting arguments) may be the ones most likely to undermine, rather than strengthen, self-efficacy. This is similar to research findings that AI-assisted writing leads to a decrease in researchers’ self-credit. High-quality AI that does things researchers value doing may produce a different psychological calculus than high-quality AI that does things they find burdensome. Future research should examine this distinction empirically.

### 5.4 Theoretical implications

This study advances theory in three ways. First, it extends the JD-R model to AI-augmented scientific work by demonstrating that the motivational and health-impairment processes can operate as parallel, complementary pathways within a single model. The finding that the loss-alleviation pathway, rather than the motivational gain pathway, carried the significant indirect effect specifies when each process dominates in technology-rich academic contexts, refining prior JD-R applications that treated digital tools as uniform resources (Lequeurre et al., 2013). Second, the asymmetric moderating role of AI trust positions trust as a threshold condition rather than a continuous amplifier of AI benefits, adding theoretical precision to the emerging literature on AI trust. Third, by introducing and concretizing the concept of cognitive verification load, a specific subtype of cognitive load related to human-AI collaborative research, this study further enriches the relevant theory. This study extends the cognitive load theory to the context of AI-assisted research.

## 6 Practical implications

### 6.1 For AI tool developers

The primacy of the cognitive offloading pathway has clear design implications. Rather than only boosting raw algorithm performance, developers should prioritize functions that directly cut repetitive verification labor and build transparent trust mechanisms to cross the trust threshold for low-trust researchers. First, embed traceable AI output modules. For example, the auto-generate source citations, calculation logic, parameter records and hallucination risk labels for all generated text, data and charts, so researchers can locate questionable content without full rechecks, largely reducing repetitive cross-validation work. Second, enhance hierarchical intelligent verification assistance tools. Lightweight one-click rationality screening can be designed for routine results, while deep risk warnings can be triggered for high-risk outputs such as statistical models and theoretical derivations. This separates low-risk and high-risk content, avoiding a uniform comprehensive check. Researchers need tools that make it easier to know when to trust outputs, not just tools that produce better outputs. Interpretability and calibrated transparency are not add-ons; they are central to the wellbeing value proposition. Third, developers should also pay attention to optimizing human-computer interaction design to reduce extraneous cognitive load caused by improper interface design. Many AI tools have overly complex interfaces, chaotic feature layouts, and unclear ways of presenting information. This not only increases the learning cost but also distracts researchers during use, consuming valuable cognitive resources. Future AI research tools should adopt a simple and intuitive interface design, highlight core functions, closely integrate verification-related functions with generation functions, and avoid unnecessary operational steps.

The trust threshold finding additionally implies that developers cannot rely on quality improvements alone to activate the cognitive-relief pathway. Calibrated trust-building mechanisms—audit trails, explanation interfaces, performance history displays—may be as important as performance improvements in translating quality into well-being gains, particularly for researchers who are currently in the low-trust zone.

### 6.2 For individual researchers

Researchers should approach AI strategically: as a tool for reducing verification overhead on well-defined, bounded tasks rather than as a competence amplifier for core intellectual work. Tasks involving heavy repetitive checking—data formatting, reference management, preliminary literature screening—are natural candidates for AI delegation. Maintaining personal ownership of hypothesis formulation, argument construction, and critical evaluation preserves both research integrity and the sources of meaning that sustain wellbeing.

Trust calibration matters. Researchers with persistently low trust may be foregoing the cognitive-relief benefits available to them from high-quality tools. Targeted familiarization with specific tools’ reliability profiles, failure modes, and appropriate use cases may help develop calibrated—rather than globally high or low—trust, enabling the threshold to be crossed for appropriate tasks without exposing researchers to the risks of overtrust on high-stakes outputs.

### 6.3 For research institutions

Traditional concepts often hold that users should be taught how to use AI more effectively, such as optimizing prompts, mastering more functions, or improving human-computer interaction efficiency. However, the results of this study point to a more practically valuable conclusion: the focus is not on teaching how to use AI, but on teaching how to efficiently verify AI outputs, thereby truly reducing cognitive verification load. Teaching researchers structured verification heuristics—rapid plausibility checks, targeted cross-validation for high-stakes claims, stratified scrutiny by task type—can substantially reduce cognitive verification load while maintaining rigorous standards. This reframes AI literacy from a capability skill to a burden-management skill. Therefore, the management should shift the focus of AI training from operational skills to verifying efficiency literacy, and develop supportive institutional mechanisms to reduce the long-term cognitive burden of researchers.

In research institutions, especially universities, we can build Ai verification standard templates shared by teams. The inspection procedures for experimental data, manuscripts and simulation results shall be unified according to the classification of disciplines. New members can follow the mature workflow and avoid repeated trial and error verification. Secondly, it is necessary to clearly formulate institutional norms on AI attribution and academic accountability, and clarify the final review responsibility of researchers for all AI generated content. And establish regular AI feedback channels to collect common verification pain points of scholars. Institutions should also attend to the identity implications of AI adoption. Policies and practices that preserve researchers’ sense of intellectual agency, framing AI as an instrument for serving the researcher’s curiosity rather than for replacing it, are likely to sustain the motivational and well-being resources that make research sustainable over the long term.

## 7 Limitations and future directions

Three limitations warrant acknowledgment. First, the cross-sectional design precludes causal inference. The mediation model assumes a temporal sequence—quality perceptions precede self-efficacy and load changes, which in turn precede well-being outcomes—that cannot be verified with concurrent measurement. Longitudinal panel designs or experience-sampling studies are needed to establish temporal precedence. An alternative causal direction is plausible: researchers with higher baseline wellbeing may rate AI quality more positively, creating reverse causation that cross-sectional data cannot rule out.

Second, all variables were assessed by self-report, introducing potential social desirability bias despite anonymous administration. Future research should triangulate self-report with objective behavioral data (logged AI usage, error detection rates) and physiological indicators of cognitive load (pupillometry, EEG) to validate the cognitive burden construct.

Third, the sample was drawn from a single national context, limiting generalizability. Given evidence that cultural factors moderate AI-wellbeing relationships (Liebherr, 2025), cross-national replications with structurally diverse research systems are warranted. Future studies should also examine additional boundary conditions—including perceived job security, discipline-level norms around AI use, and career stage—that may shift the relative strength of gain and loss pathways.

Fourth, our recruitment strategy combining online academic groups and snowball sampling carries inherent potential selection bias. Researchers with strong positive or negative attitudes toward AI may be disproportionately willing to complete the survey, while scholars who rarely adopt AI tools are likely underrepresented in the sample. In future studies, we’ll be careful in choosing samples to make them more representative.

Fifth, the study suffers from temporal limitations driven by rapidly evolving AI technology. Generative AI functions, reliability, interpretability, and academic regulatory standards shift continuously. The perceptions captured in this snapshot sample may become outdated as more advanced, transparent AI research tools emerge. Repeated time-series surveys are necessary to track shifting associations between AI quality, trust and well-being as technology matures.

## 8 Conclusion

The question that motivated this study—does better AI actually improve researcher well-being?—turns out to have a conditional, mechanistic answer. Better AI improves wellbeing primarily by reducing the cognitive burden of verification, not by making researchers feel more scientifically capable. And even this cognitive-relief benefit requires a minimum level of trust to activate: high-quality AI is effectively invisible to researchers who cannot bring themselves to rely on it. These findings carry a practical message: the path from AI quality to researcher well-being runs through trust and cognitive burden, not through competence. Developers who optimize for transparency, interpretability, and calibrated reliability, rather than raw capability, are more likely to produce tools that improve the lived experience of research. The value of AI in science is not that it thinks better, but that it allows researchers to think more freely.

## Data Availability

The raw data supporting the conclusions of this article will be made available by the authors, without undue reservation.
