# Supplementary material for: The impact of AI service quality on researchers’ work wellbeing: a dual-path moderated mediation model
Source: Front Psychol. 2026 Oct 16;17:1946487. doi: 10.3389/fpsyg.2026.1946487 (PMC13719630; doi:10.3389/fpsyg.2026.1946487)
Supplement: Supplementary file 1 [file Supplementary_file_1.docx]

**Appendix**

| **Code** | **English wording** | **Chinese wording** |
| --- | --- | --- |
| AI Service Quality | 1. The AISA works correctly at first attempt. | 1.我认为AI提供的内容是准确的 |
|  | 2. Using the AISA is fun. | 2.我认为AI提供的内容是丰富有趣的 |
|  | 3. The AISA adequately meets my requirements. | 3.我认为AI提供的内容与我的科研需求匹配 |
|  | 4. The AISA is never too busy to respond to my requests. | 4.AI不会因繁忙而无法响应我的请求 |
|  | 5. The AISA interface design provides information clearly. | 5.我发现AI平台很容易使用 |
|  | 6. The AISA is able to communicate like a human. | 6.我与AI的交流与真人一样清晰易懂 |
|  | 7. The AISA is always available. | 7.我在科研工作中经常性地使用AI |
|  | 8. The time I spent on the AISA was worthwhile. | 8.AI提供的内容有助于我解决科研问题 |
|  | 9. I will use the AISA more in the coming months. | 9.我会继续使用AI为我提供科研帮助 |
| AI Trust | 1. I trust generative‑AI when doing academic‑related work. | 1.在做科研工作时，我都相信AI |
|  | 2. I do not trust generative‑AI when doing academic‑related work. | 2.在做科研工作时，我不相信AI |
|  | 3. I rely on generative AI for my academic tasks. | 3.一般来说，在做科研工作时，我会依赖AI |
|  | 4. I have a general tendency to trust generative AI. | 4.我有强烈的信任AI的倾向 |
|  | 5. I readily trust generative AI to complete my academic tasks. | 5.我很容易信任AI来执行科研任务 |
|  | 6. I may trust generative AI even with limited knowledge about it. | 6.当我对AI了解不多时，我仍然可能信任它 |
|  | 7. I believe my data entered into generative AI will remain secure. | 7.我认为在AI中输入信息不会被泄露 |
|  | 8. I can trust information outputs provided by generative AI. | 8.我可以信任从AI中获得的信息 |
|  | 9. If I trust generative AI outputs, I do not need to cross‑check its content. | 9.我信任来自AI的信息，因此我不必去核实 |
| Research Self-Efficacy | 1. Difficulty perception for learning novel research tools. | 1.对我来说，学习使用新的AI很难 |
|  | 2. Uncertainty about one’s own research competence. | 2.我对使用AI辅助科研的能力感到不确定 |
|  | 3. Frequent difficulties when acquiring new research‑relevant skills. | 3.我经常在学习使用AI时遇到困难 |
|  | 4. Perceived complexity of new research‑related tools. | 4.AI技术对我来说太复杂了 |
|  | 5. Perceived ease of performing research‑related tasks. | 5.我觉得使用AI辅助科研很容易 |
|  | 6. Confidence in mastering new research‑relevant techniques. | 6.我非常确信自己在掌握新AI技术方面的能力 |
|  | 7. Proficiently carry out routine research activities. | 7.我可以熟练地使用AI帮助我完成日常科研工作 |
|  | 8. Belief that one can boost one’s own research productivity. | 8.我可以使用AI来提高我的科研工作效率 |
|  | 9. Critical evaluation of research tools; recognize tool strengths and weaknesses. | 9.我可以评估一个AI工具的能力和局限性 |
|  | 10. Select appropriate approaches for solving research problems. | 10.我可以从AI提供的各种解决方案中选择一个合适的方案 |
|  | 11. Match methods/tools to given research tasks. | 11.我可以为特定科研任务选择最合适的AI产品 |
| Cognitive Verification Load | 1. I can easily identify false information in AI generated content. | 1.我能轻松识别AI生成内容里的虚假信息 |
|  | 2. I can easily determine whether AI generated content is reliable. | 2.我可以很轻松地判断AI生成内容是否可靠 |
|  | 3. I can easily identify advertising information in AI generated content. | 3.我可以很容易识别出AI生成内容中的广告信息 |
|  | 4. When I use AI to obtain useful information, I can directly find the original literature for verification. | 4.当我用AI获取到有用的信息时，我能直接找到原始文献进行核实 |
| Work Well-Being | 1. At my work, I feel bursting with energy. | 1.在科研工作中，我感到精力充沛 |
|  | 2. At my job, I feel strong and vigorous. | 2.科研工作时，我感到自己很强大且充满活力 |
|  | 3. When I get up in the morning, I feel like going to work. | 3.我为自己的工作感到自豪 |
|  | 4. When I am working, I forget everything else around me. | 4.当我全身心投入工作时，我会感到非常开心 |
|  | 5. Time flies when I am working. | 5.我沉浸于我的工作当中 |
|  | 6. I get carried away when I am working. | 6.我在工作时会达到忘我的境界 |
|  | 7. When I get up in the morning, I feel like going to work. | 7.早上一起床，我就想要去工作 |
|  | 8. I am enthusiastic about my job. | 8.我对自己的工作充满热情 |
|  | 9. My job inspires me. | 9.我的工作给了我极大的动力 |

*Note. For each scale, the English column presents the original English item wording as published in the source scale, and the Chinese column presents the adapted, administered version used in this study. Among them, the original text measuring Research Self-Efficacy did not report the complete items, so only the original construction prototypes from the reference are shown here. The items for measuring Cognitive Verification Load are based on the scale paradigm from the original literature, rather than directly adapting its existing items.*
